# An enzyme-based system for extraction of small extracellular vesicles from plants

**DOI:** 10.1038/s41598-023-41224-z

**Published:** 2023-08-25

**Authors:** Qing Zhao, Guilong Liu, Fubin Liu, Manlin Xie, Yanfang Zou, Shengpeng Wang, Zhaodi Guo, Jiaming Dong, Jiali Ye, Yue Cao, Lei Zheng, Kewei Zhao

**Affiliations:** 1 Guangzhou Key Laboratory of Chinese Medicine Research on Prevention and Treatment of Osteoporosis, The Third Affiliated Hospital of Guangzhou University of Chinese Medicine, Guangzhou, 510378 Guangdong China; 2 The Third Clinical Medical School of Guangzhou University of Chinese Medicine, Guangzhou, 510403 Guangdong China; 3 Department of Blood Transfusion, Sun Yat-sen Memorial Hospital, Sun Yat-sen University, Guangzhou, 510378 Guangdong China; 4 State Key Laboratory of Quality Research in Chinese Medicine, University of Macau, Macao, 519000 China

**Keywords:** Isolation, separation and purification, Biological techniques, Plant sciences

## Abstract

Plant-derived nanovesicles (NVs) and extracellular vesicles (EVs) are the next generation of nanocarrier platforms for biotherapeutics and drug delivery. EVs exist not only in the extracellular space, but also within the cell wall. Due to the limitations of existing isolation methods, the EVs extraction efficiency is low, and a large amount of plant material is wasted, which is of concern for rare and expensive medicinal plants. We proposed and validated a novel method for isolation of plant EVs by enzyme degradation of the plant cell wall to release the EVs. The released EVs can easily be collected. The new method was used for extraction of EVs from the roots of *Morinda officinalis* (MOEVs). For comparison, nanoparticles from the roots (MONVs) were extracted using the grinding method. The new method yielded a greater amount of MOEVs, and the vesicles had a smaller diameter compared to MONVs. Both MOEVs and MONVs were readily absorbed by endothelial cells without cytotoxic effect and promoted the expression of miR-155. The promotion of miR-155 by MOEVs was dose-dependent. More importantly, we found that MOEVs and MONVs were enriched toward bone tissue. These results support our hypothesis that EVs in plants could be efficiently extracted by enzymatic cell wall digestion and confirm the potential of MOEVs as therapeutic agents and drug carriers.

## Introduction

Nanovesicles (NVs) isolated from plants are structurally similar to mammalian exosomes^1^ and can regulate biological functions across membranes in animals and humans^2–6^**.** Several recent studies have shown that plant-derived NVs have intrinsic therapeutic activities such as maintaining intestinal stem cells and shaping the intestinal microbiota to enhance intestinal barrier function and relieve colitis^7^, improving anti-inflammatory properties in intestinal diseases^8^, preventing alcohol-induced liver injury^9^, promoting wound healing^10^, and participating in tumor immunomodulation^3^. In addition, these vesicles have drug delivery properties. For example, Wang et al. found that loading methotrexate (MTX) into grapefruit-derived NVs significantly reduced MTX toxicity and significantly improved its therapeutic effect on dextran sodium sulfate-induced colitis in mice^11^. Plant-derived NVs are considered potential therapeutic agents or drug carriers due to their small size and low immunogenicity. Compared to mammal NVs, they are available from a wide range of medicinal plant sources, do not carry human or zoonotic pathogens, and have unique therapeutic activities^12^.

In mammals, extracellular vesicles (EVs) can be directly extracted from organic liquids. In contrast, in plants, EV structures exist in the extracellular space and within the cell wall^13^. Two main methods have been developed for the extraction of plant-derived NVs and EVs, grinding and apoplastic fluid extraction by vacuum infiltration^14^**.** Grinding is the most commonly used method^8, 15, 16^ that yields large amounts of NVs. However, since these vesicles are derived from whole plant tissues subjected to cellular destruction, a mixture of vesicles uneven in size and activity are extracted in addition to pure EVs, thus influencing the results of studies. The apoplastic fluid extraction by vacuum infiltration^14^ produces only a few EVs due to the cell wall barrier effect, which restricts the passage of EVs. This low extraction efficiency results in the waste of a large amount of plant raw material, and the method does not meet the requirements of mass preparation. Therefore, the development of a more efficient separation method is critical for the study of plant derived EVs.

We proposed a new pretreatment method for separation of plant derived EVs. The plant cell walls were degraded with digestive enzymes targeting the main wall components, which not only promoted the release of EVs within the cell wall, but also facilitated the release of EVs from the cell wall into the surrounding solution. The method improves the output of isolated EVs, and more importantly, reduces contamination by intracellular components. The method has been validated in the extraction of EVs from *Morinda officinalis* (MO) roots.

**Figure 1:**
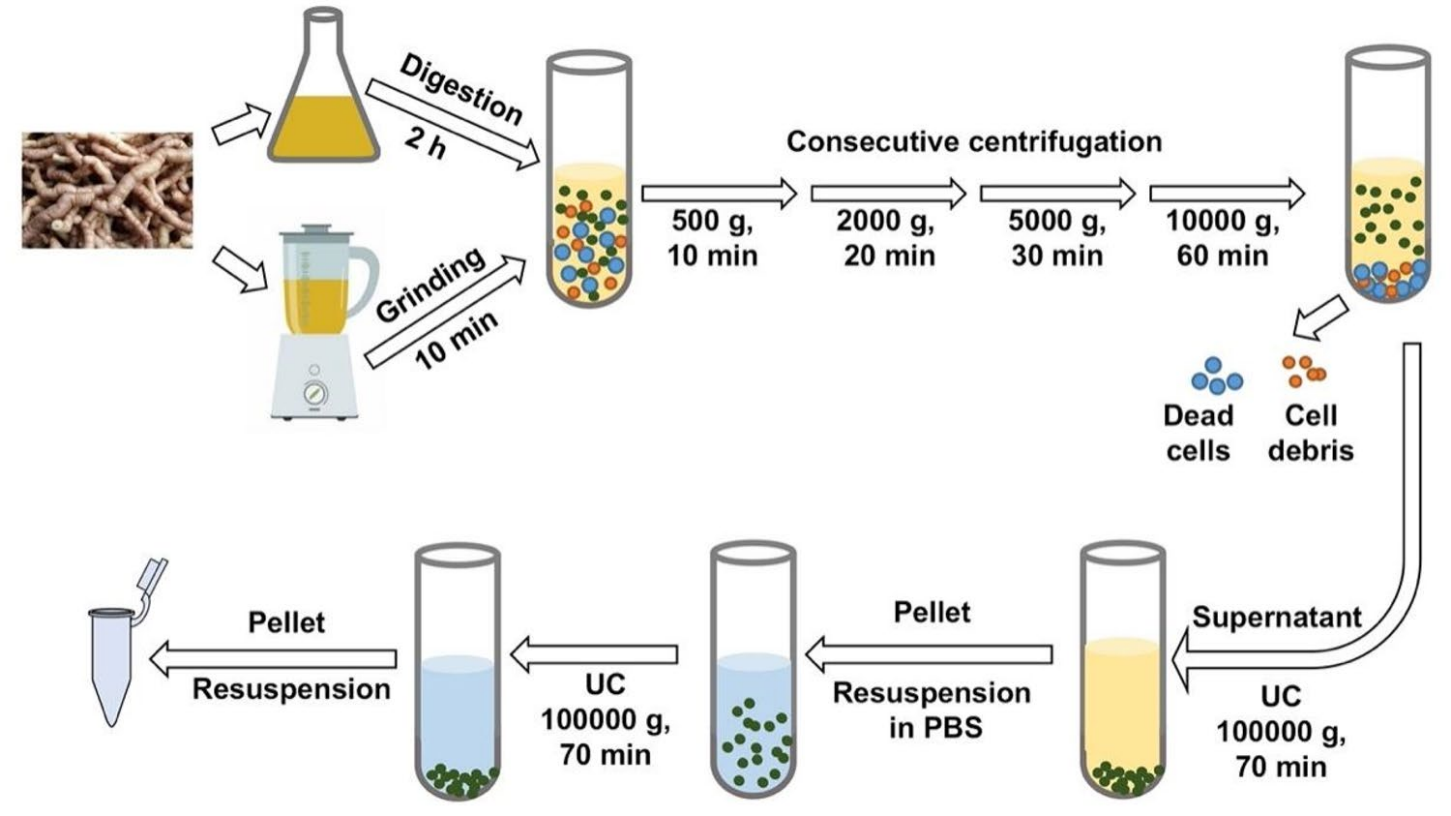
Scheme for isolation and preparation of MOEVs/MONVs.

*Morinda officinalis* is one of the four popular medicinal plants in South China. The root of MO, as a known medicinal ingredient in Traditional Chinese medicine, has a variety of biological activities and functions, including anti-osteoporotic^17–19^, anti-inflammatory^20–22^, and antioxidative activity^23^. In the present study, enzyme digestion and grinding were used to isolate EVs from the root of MO. The EVs extracted by enzyme digestion were named MO-derived EVs (MOEVs), and the NVs extracted by the grinding process were named MO-derived nanoparticles (MONVs) (Fig. 1). We compared MOEVs and MONVs using multiple aspects to further verify the effectiveness of enzymatic isolation of plant derived EVs.

## Results

### Characterization and yield of MONVs and MOEVs

**Figure 2:**
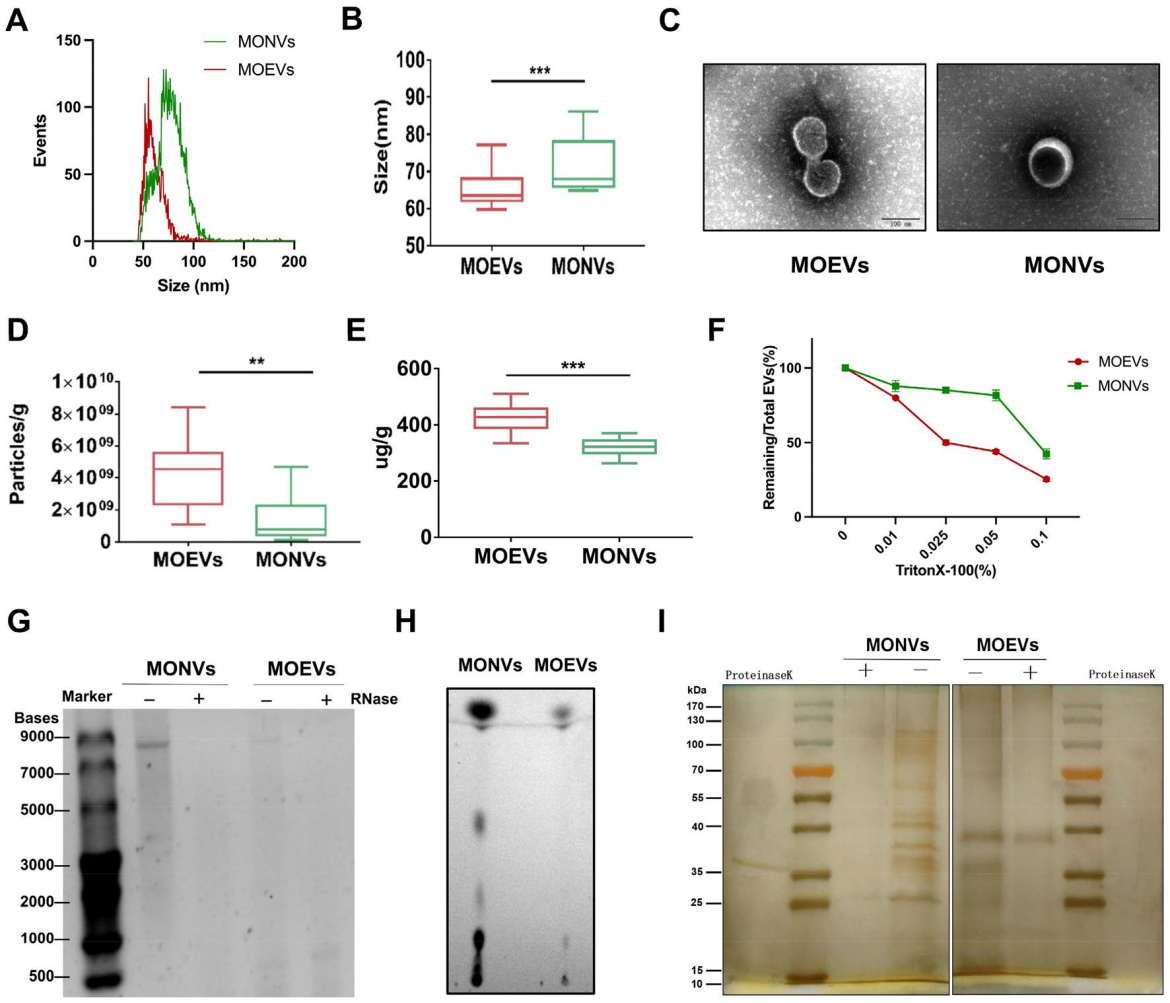
Characterization and yield of MONVs and MOEVs. (**A, B**) Diameter distribution of MONVs and MOEVs by nano-flow detection. (**C**) Morphology of MONVs and MOEVs analyzed by TEM. (**D**) Particle yields and (**E**) protein yields of MONVs and MOEVs. (**F**) Triton X-100 membrane breaking experiment. (**G**) RNA by 2.5% agarose gel electrophoresis, (**H**) Lipid gel electrophoresis, and (**I**) Protein gel electrophoresis of MOEVs and MONVs. All values are expressed as mean ± SD (**p < 0.01, ***p < 0.001; *n* = 9).

To determine the optimum enzyme reaction concentration and reaction time, we set three concentrations and three times for screening using nano-flow cytometry *(*nanoFCM). The results suggest that the best concentration of MOEVs protein was obtained by incubating plant tissues in 0.2% of the enzyme with bacitracin for 2 h (Supplementary Fig. 1). Most MONVs had a diameter in the range of 50–120 nm, with an average peak diameter of 71.62 ± 2.296 nm (*n* = 9) (Fig. 2A and B). Most MOEVs had a diameter in the range of 50–80 nm, with an average peak diameter of 65.46 ± 1.74 nm (*n* = 9) (Fig. 2A and B). MOEVs had a smaller size range and a more uniform diameter distribution compared to MONVs. Transmission electron microscopy (TEM) images corroborated the typical exosome-like morphology of both MONVs and MOEVs (Fig. 2C). The yield of MONVs and MOEVs were (1.41 ± 0.52) × 10^9^ particles and (4.35 ± 0.74) × 10^9^ particles per gram of tissue, respectively (Fig. 2D). Protein content of MONVs (322.2 ± 10.51 µg/g tissue) was lower than that of MOEVs (423.8 ± 17.45 µg/g tissue) (Fig. 2E).

To indicate the purity of EVs, we performed membrane solubilization using Triton X-100, a surfactant that disrupts membrane integrity. The particle concentration of EVs gradually decreased with increasing Triton X-100 concentration, and compared with an absence of TritonX-100, the number of particles when treated with 0.5% TritonX-100 decreased significantly to 28% (Fig. 2F). Therefore, we could reliably isolate abundant MONVs and MOEVs from *Morinda officinalis*.

To further elucidate the main chemical composition of MONVs and MOEVs, they were analyzed for lipid, RNA, and protein content by thin-layer liquid chromatography (TLC), 2.5% agarose gel electrophoresis and PAGE gel electrophoresis, respectively. The amount of lipids and RNA was much higher in MONVs than in MOEVs (Fig. 2G and H). The RNA content in MONVs and MOEVs was altered by RNase, suggesting that these vesicles contain RNA (Fig. 2G). The number of protein types in MOEVs were significantly greater than those in MONVs for the same total amount of protein (20 μg), and the majority of the proteins are degraded after the addition of proteinase K (Fig. 2I). These results suggest that the compositions of MONVs and MOEVs differ, which may be related to their different origins. These results suggested differential properties between MONVs and MOEVs.

### Effects of MONVs and MOEVs on efficiency of vascular endothelial cells (ECs) uptake

**Figure 3:**
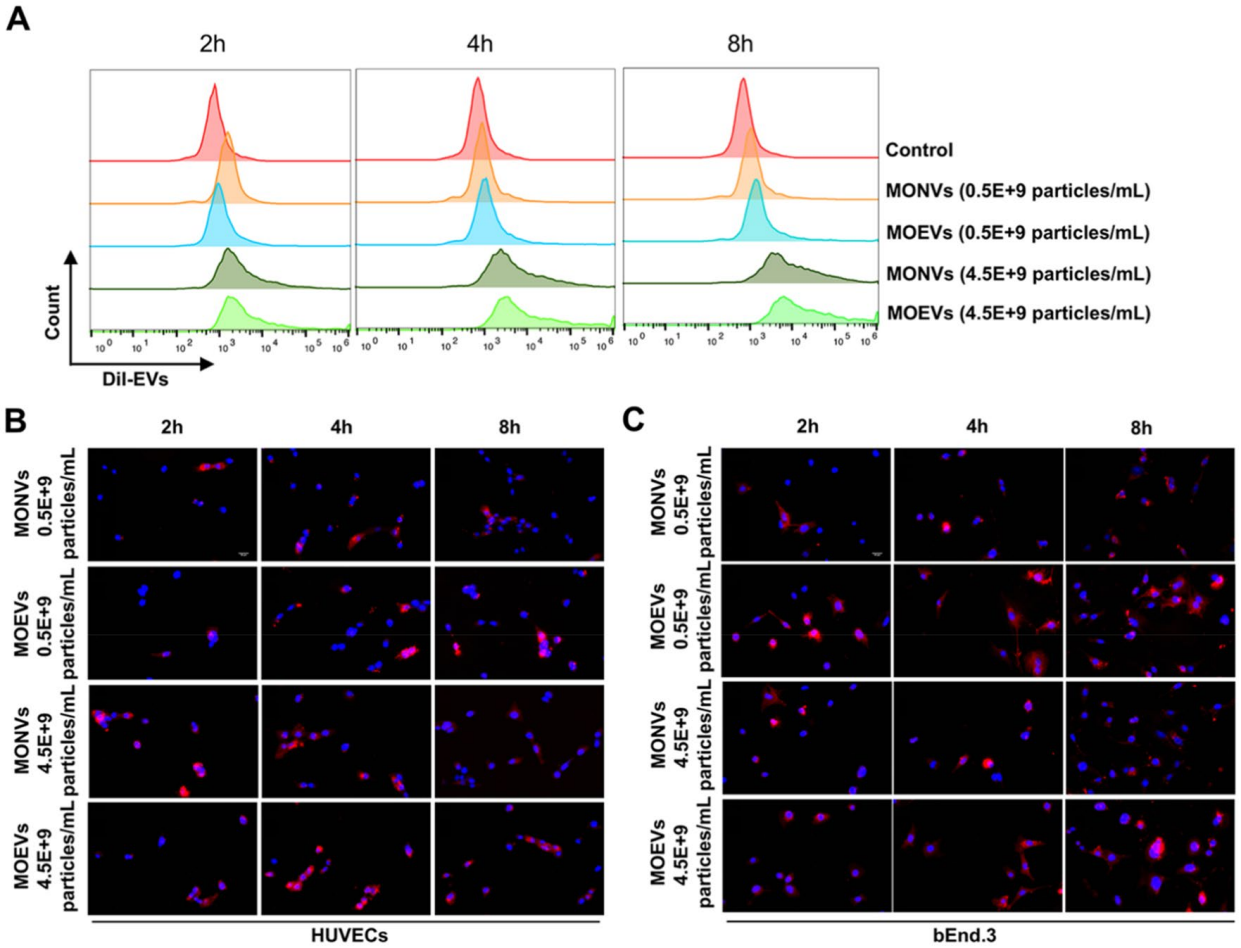
Time profile of in vitro uptake of different concentrations of MONVs and MOEVs by bEnd.3 and h-UVECs. (**A**) Flow cytometry assays about Dil-labeled MONVs and MOEVs of different concentrations (0.5 × 10^9^ particles/ml, 4.5 × 10^9^ particles/mL) uptaken by h-UVECs. Fluorescent microscopic images of Dil-labeled MONVs and MOEVs of different concentrations (0.5 × 10^9^ particles/ml, 4.5 × 10^9^ particles/mL) uptaken by h-UVECs (**B**) and bEnd.3 (**C**). The Control was a blank control cell. MONVs and MOEVs were labeled with DiI in red, and the nuclei were labeled with Hoechst33342 in blue. (Scale bar: 50 μm, *n* = 3).

Blood vessels are closely associated with osteogenesis. To determine whether MONVs and MOEVs differentially affect mammalian cell uptake capacity, we compared the efficiency of vesicle uptake using a mouse brain-derived endothelial cell line (bEnd.3) and human umbilical vein endothelial cells (h-UVECs). MONVs and MOEVs were labeled with fluorescent lipophilic 1,1′-dioctadecyl-3,3,3′,3′tetra- methylindocarbocyanine perchlorate (DiI) and then co-incubated with bEnd.3 and h-UVECs. MONVs and MOEVs (red) were rapidly taken up by bEnd.3 and h-UVECs and preferentially localized in the cytoplasm of the cells (Fig. 3B and C). Flow cytometry indicated that the percentage of h-UVECs that uptake DiI-MONVs and DiI-MOEVs increased with time and dose (Fig. 3A). The percentage of h-UVECs that uptake MONVs and MOEVs increased from 2.62% and 5.73% at low concentrations to 24% and 35.6% at high concentrations after 2 h, respectively. The percentage of h-UVECs ingesting MONVs and MOEVs increased from 4.78% and 11.20%, respectively, at low concentrations to 55.4% and 75.7%, respectively, at high concentrations after 8 h. Both MONVs and MOEVs were readily taken up by ECs, although the uptake efficiency of MOEVs was higher, which may be related to their smaller particle size (Fig. 2A and B).

### MONVs and MOEVs had no cytotoxicity and promoted the expression of miR-155 in ECs

**Figure 4:**
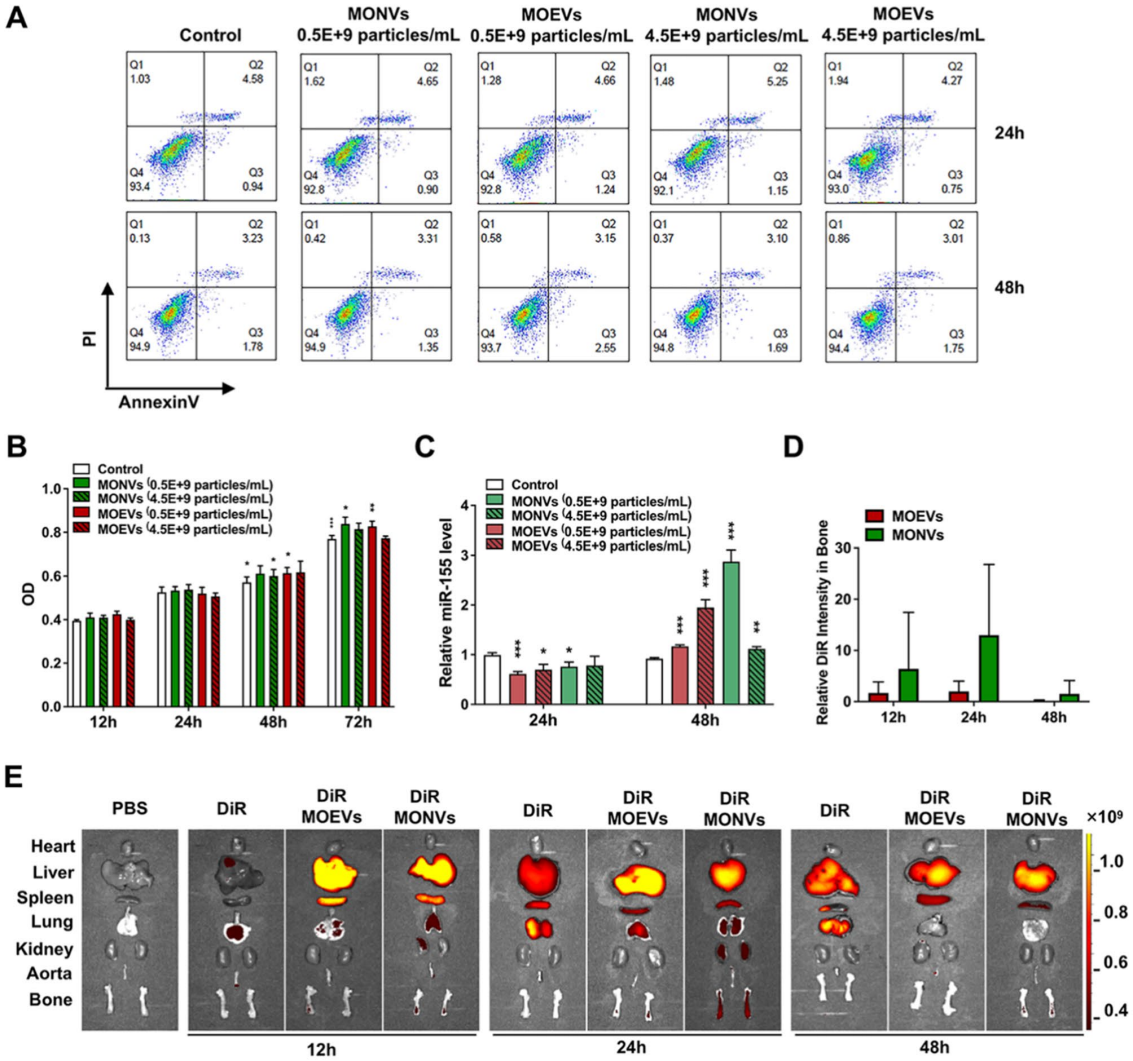
Effect of MOEVs and MONVs on the Proliferation, activity, and promotion of miR-155 expression in h-UVECs. (**A**) Effect of different concentrations of MOEVs and MONVs on the activity of h-UVECs detected by flow cytometry. (**B**) CCK8 assay of the effect of different concentrations of MOEVs and MONVs on the Proliferation of h-UVECs. (**C**) RT-PCR histogram of the effect of different concentrations of MOEVs and MONVs on miR-155 expression in h-UVECs. In vivo organ distribution of MOEVs and MONVs. (**D**) Relative fluorescence intensity histograms of bone tissue at different times, with free DiR as the control group. (**E**) In vivo organ distribution of MOEVs and MONVs in mice.

We evaluated the toxicity of both vesicles to cells and their function on endothelial cells in vitro. The co-incubation of MONVs and MOEVs at different concentrations with h-UVECs showed no significant cytotoxicity within 72 h. h-UVECs still maintained > 90% viability at 24 h and 48 h co-incubation (Fig. 4A). Low concentrations (0.5 × 10^9^/mL) of MONVs and MOEVs significantly promoted the proliferation of h-UVECs at 48 h and 72 h, whereas high concentrations (4.5 × 10^9^/mL) of MONVs and MOEVs showed different performance results (Fig. 4B). A high concentration of MOEVs did not significantly promote the proliferation of h-UVECs, whereas a high concentration of MONVs still significantly promoted the proliferation of h-UVECs at 48 h and 72 h (Fig. 4B). The results suggest that both MONVs and MOEVs are not cytotoxic and promote EC proliferation.

miR-155 regulates neovascularization and osteogenic differentiation. We next analyzed the expression levels of miR-155 in h-UVECs co-incubated with MONVs or MOEVs for 24 or 48 h. MONVs and MOEVs both slightly inhibited the expression of miR-155 and were negatively correlated with the level of miR-155 at 24 h. Generally, MONVs and MOEVs were similar in their efficacy to regulate the expression of miR-155 in h-UVECs, but MOEVs efficiency was dependent on the concentration at 48 h (Fig. 4C).

### Bone targeting ability of MONVs and MOEVs

Due to targeted therapy is the main method to reduce adverse drug reactions, we evaluated the bone-targeting ability of MONVs and MOEVs. When mice were injected with 1,1-dioctadecyl-3,3,3,3-tetramethylindotricarbocyanine iodide (DiR) labeled MONVs and MOEVs in the tail vein for 12 and 24 h, we observed strong fluorescent signals (Fig. 4E). The fluorescence signals of MOEVs almost disappeared, whereas strong fluorescence signals of MONVs could still be detected at 48 h in bone (Fig. 4D and E). In general, both MONVs and MOEVs were capable of bone targeting, and the fluorescence intensity changed over time (Fig. 4D and E).

### Liquid chromatograph-mass spectrometer (LC–MS) metabolomics of MONVs and MOEVs

**Figure 5:**
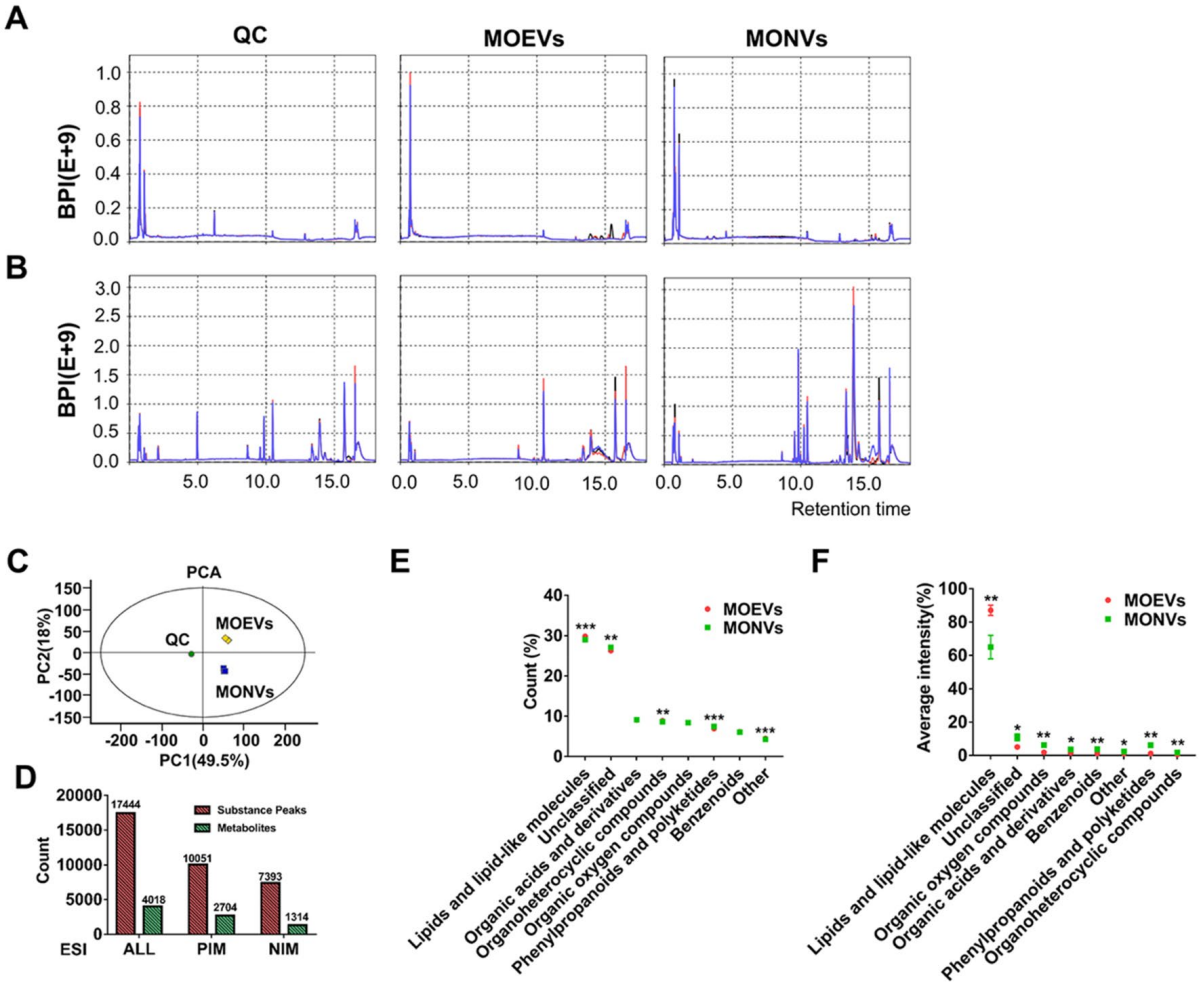
Quality control and substance confirmation of chromatographic-mass spectrometric detection of MOEVs and MONVs contents. (**A, B**) Base peaks of QC, MONVs, and MOEVs in negative (**A**) and positive (**B**) ion modes. (**C**) Dot plots of PCA scores of QCs, MONVs, and MOEVs. (**D**) Histogram of the number of peaks and substance confirmation for MONVs and MOEV in the negative (NIM) and positive ion modes (PIM). (**E, F**) Composition of MONVs and MOEVs compounds. (**E**) Percentage of compound classification counts and (**F**) intensity.

To initially explore the metabolic components that may play a role in vesicles, we performed a non-targeted metabolomics assay for vesicle analysis. The results of untargeted metabolomics displayed a regular and uncluttered basal peak pattern, and the QC samples in the PCA model diagram (obtained through seven cycles of cross-verification) were closely clustered together, which proved that the instrument was stable, and the collected data was reliable (Fig. 5A–C). A total of 17,444 chromatographic peaks were extracted from MONVs and MOEVs, comprising 10,051 positive ion mode (PIM) chromatographic peaks and 7393 negative ion mode (NIM) chromatographic peaks. The comparison of the peaks with the related database identified 4018 compounds, namely 2704 in PIM and 1314 in NIM modes (Fig. 5D). Classification of 4018 identified compounds showed that MONVs and MOEVs included similar species of compounds; the main types included lipids and potentially functional molecules (29.76% vs 28.91%, respectively), organic oxygen compounds (9.22% vs. 8.95%, respectively), and organic acids and derivatives (8.81% vs. 8.59%, respectively) (Fig. 5E). In addition, 27.35% and 26.15% of compounds in MONVs and MOEVs, respectively, were not classified (Fig. 5E). The relative content of the above compounds differed between MONVs and MOEVs. The content of lipids and lipid-like molecules was significantly higher in MOEVs than in MONVs (87.62% vs. 66.17%, respectively; *p* < 0.01) (Fig. 5F).

**Figure 6:**
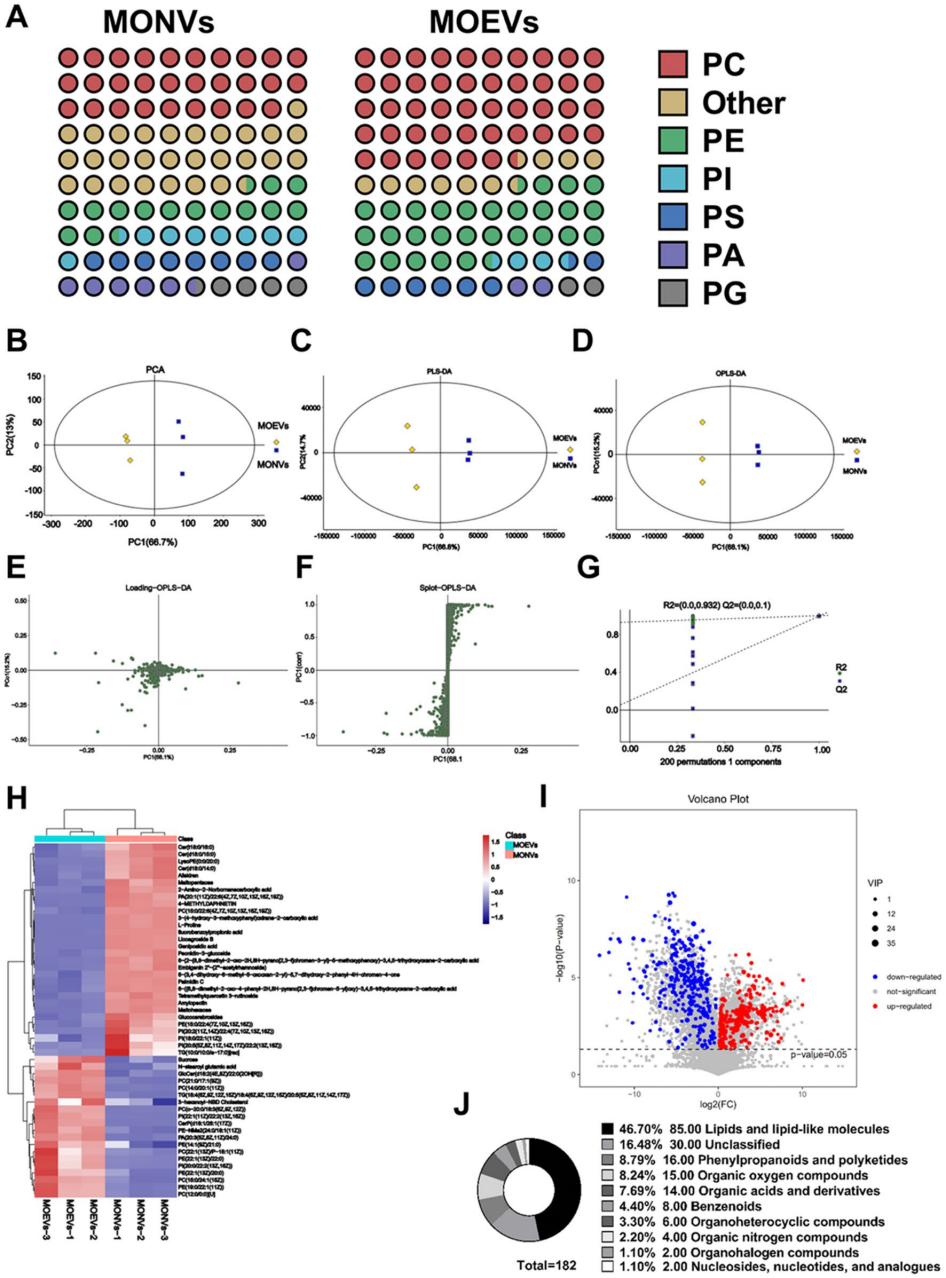
Multivariate statistical analysis of lipid composition and compounds of MONVs and MOEVs. (**A**) Pie chart of lipid content. PC = phosphatidylcholine, PS = phosphatidylserine, PE = phosphatidylethanolamine, PI = phosphatidylinositol, PA = phosphatidic acid, PG = phosphatidylglycerol. (**B**) Plot of PCA scores of MONVs and MOEVs. (**C**) Plot of MONVs and MOEVs PLS-DA scores. (**D**) MONVs and MOEVs OPLS-DA score plots. (**E**) Permutation plot. (**F**) Loading plot. (**G**) S-plot. Differential metabolite screening. (**H**) Heat map of top 50 differential metabolites. (**I**) Volcano plot of VIP and P screens for differential metabolites. (**J**) Pie chart of differential metabolites.

Among the lipids in MONVs, phosphatidylcholine (PC) was the most abundant (29.18%), followed by phosphatidylethanolamine (PE; 14.86%) and phosphatidylinositol (PI; 8.53%) (Fig. 6A). In MOEVs, PC accounted for 46.4% of the lipids, 1.6 times higher than that in MONVs, followed by PE (29.06%) and phosphatidylserine (PS; 7.71%) (Fig. 6B). Similarly, the content of other lipids significantly differed between MONVs and MOEVs (28.52% vs. 9.92%, respectively; *p* < 0.01) (Fig. 6A). Unsupervised principal component analysis (PCA) (R^2^X = 0.806) could accurately distinguish between MONVs and MOEVs, and the whole analysis process was stable and reliable (Fig. 6B). Supervised partial least-squares discriminant analysis (PLS-DA) similarly completely separated MONVs and MOEVs (R^2^X_CUM_ = 0.907, R^2^Y_CUM_ = 1, Q^2^_CUM_ = 0.995), indicating that the model had reliable stability and strong predictive ability (Fig. 6C). Similar results for MONVs and MOEVs were obtained by orthogonal partial least-squares discriminant analysis (OPLS-DA) (Fig. 6D). The OPLS-DA model was tested 200 times for response sequencing, and the corresponding OPLS-DA model (R^2^ = 0.932 and Q^2^ = 0.1 of the random model) was of good quality (Fig. 6E). The effects of metabolites on MONVs and MOEVs were visualized on the load diagram (Fig. 6F), and the contribution rate of each variable to the grouping of MONVs and MOEVs was illustrated by the S-plot (Fig. 6G). The model parameters of the comparative analysis of the two groups are shown in Table 1. These analyses show that the model was reliable and could be used to screen differential metabolites between MONVs and MOEVs.

**Table 1:** The model parameters of the comparative analysis of the two groups.

Group	Type	PRE	ORT	N	R2X (cum)	R2Y (cum)	Q2 (cum)	R2	Q2
C/A	PCA	3	0	6	0.806				
C/A	PLS	3	0	6	0.907	1	0.995		
C/A	OPLS	1	2	6	0.907	1	0.995	0.932	0.1

### Differential metabolite analysis

**Figure 7:**
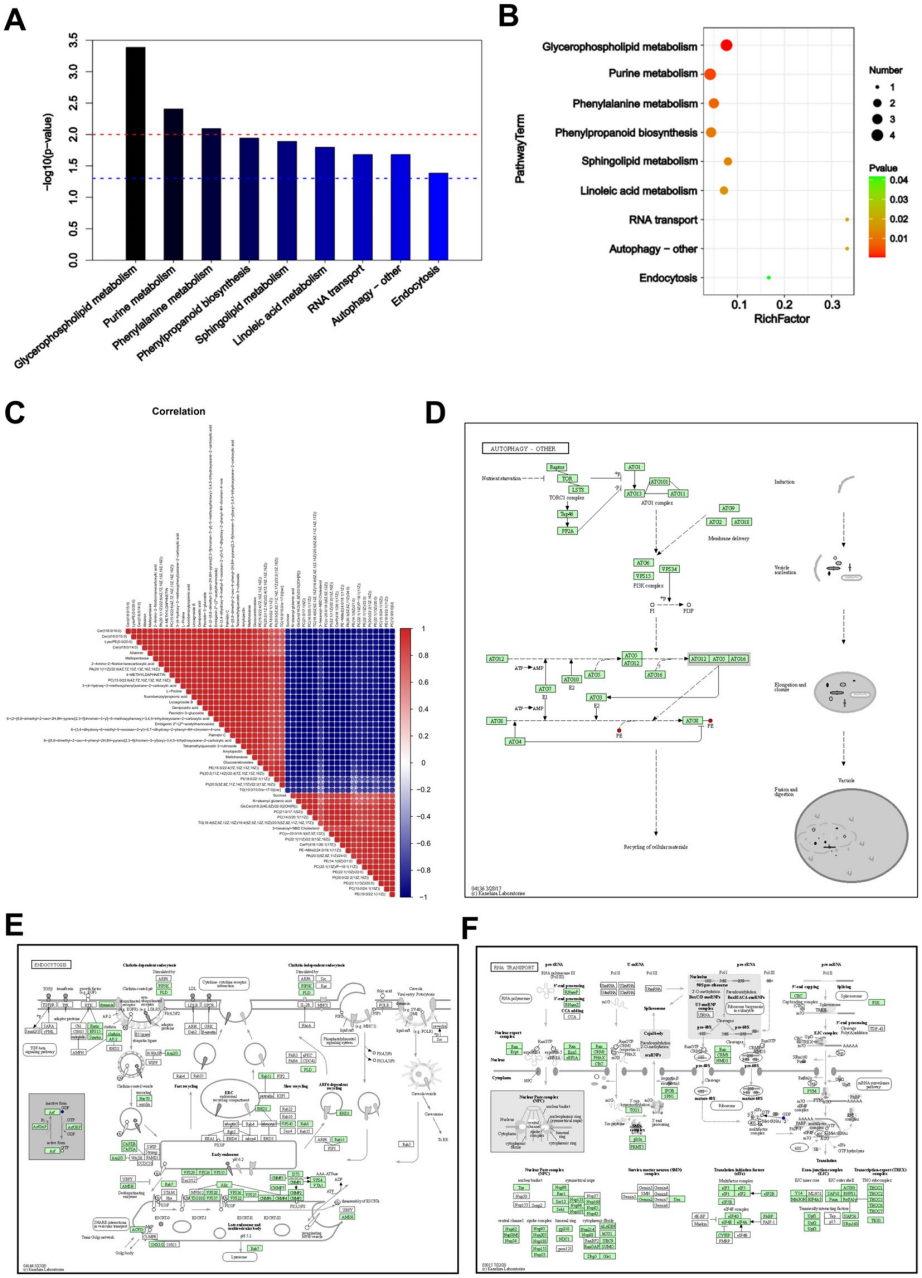
KEGG enrichment metabolic pathway analysis of differential metabolites. (**A**) Bar graph and (**B**) bubble diagram of metabolic pathway enrichment by p-value screening. (**C**) Correlation analysis of top 50 differential metabolites. Visualization of (**D**) endocytosis, (**E**) autophagy–other, and (**F**) RNA transport metabolic pathways (Open access licence of KEGG has been granted).

The p-value, variable importance at projection (VIP), and fold change values obtained by the OPLS-DA model were visualized by volcanic map to screen differential metabolites. A total of 182 compounds with significant differences were screened according to preset rules. Compared with MONVs, 114 metabolites were down-regulated, and 68 metabolites were up-regulated in MOEVs. The top five compounds with the highest VIP values, PE (19:0/children of z; 11), PC (15:0/24:1 [z]; 15), PC (0–20:0/and [z] 9 z, 6 z, 12), PE (children of 13 [z]/22:0), and PE (children of 13 [z]/20:0), were lipids (Fig. 6H). To display the relationship and the expression differences of metabolites between different samples, we performed hierarchical clustering of all significantly differentiated metabolites and the expression levels of the top 50 differentiated metabolites based on VIP values (Fig. 6H). The differential compounds were mainly lipids and functional-like molecules (46.7%), followed by phenylpropanoids and polyketides (8.79%), and organic oxygen compounds (8.24%) (Fig. 6I). Correlation analysis measures the degree of correlation between significantly different metabolites and further elucidates the relationship between metabolites in the process of biological state change (Fig. 6J). The top 50 differential metabolites were selected for visual analysis. EVs were positively correlated with lipids and negatively correlated with other compounds, suggesting that the differential compounds of EVs are regulated by lipids (Fig. 7C).

### Kyoto Encyclopedia of Genes and Genomes (KEGG) analysis

To initially explore metabolic pathways that may play a role in vesicles, we performed KEGG analysis. The 182 significantly different metabolites were enriched in 36 pathways, of which 9 pathways were significant (*p*-value ≤ 0.05). These were linoleic acid metabolism, RNA transport, autophagy–other, endocytosis, phenylalanine metabolism, phenylpropanoid biosynthesis, sphingolipid metabolism, purine metabolism, and glycerophospholipid metabolism (Fig. 7A). Further bubble plots of the significantly enriched pathways were created (Fig. 7B). The vertical coordinates are the metabolic pathway names, and the horizontal coordinates are the enrichment factors.

The differential metabolic pathways are displayed by the KEGG pathway mapper function, and the differential metabolites are colored according to the up- and downregulation information. The small circles in the metabolic pathway map represent metabolites. Metabolites marked in red in the pathway diagram are experimentally detected as upregulated metabolites and blue are downregulated metabolites. We visualized three metabolic pathway maps for RNA transport, autophagy–other, and endocytosis (Fig. 7D–F) (Open access license of KEGG has been granted). Autophagy is a degradative pathway for the removal of cytoplasmic materials in eukaryotic cells and is characterized by the formation of a double-membrane structure called the autophagosome, either in a housekeeping capacity or during stress and senescence. Autophagy–other pathway is for other eukaryotes including plants and protists, where autophagy related genes play similar roles in the life cycle.

## Discussion

Obtaining a large number of plants EVs is challenging due to the limitations of isolation methods. Therefore, the study on plant vesicles, especially the function of plant based EVs, lags behind the study of mammalian EVs. Several studies have shown that organisms with cell wall, such as plants, fungi, Gram-positive bacteria, and mycobacteria, produce EVs that are able to traverse the cell wall^28–30^. The cell wall is a thick, tough structure located outside the cell membrane composed of fibrillar and matrix components that protects the cell. It was assumed that the cell wall prevents the passage of large structures such as EVs. Several theories describe the escape of EVs through the cell wall; for example, swelling pressure may force EVs through the cell wall, organisms release EVs by regulating cell wall thickness, pore size or integrity, or EVs stimulate cell wall remodeling and thereby the release of EVs. However, the approaches are very energy intensive and difficult to implement. Therefore, we believe that only a fraction of EVs is extracted from plant protoplast fluid. Plant EVs are released extracellularly by fusion of multivesicular bodies with the plasma membrane^31^, where only a small fraction of extracellular vesicles can pass through due to the barrier effect of the cell wall. Therefore, we degraded the cell wall using a modified method for protoplast preparation to extract MOEVs. For comparison, we extracted MONVs using the grinding method. Particles larger than 150 nm are readily absorbed by the liver and spleen, and are unlikely candidates for targeted therapy^32–34^. Therefore, we used 0.22 μm membranes and 10,000 × *g* high-speed centrifugation to remove large vesicles during the isolation and purification process.

In this study, we developed a method for EVs extraction based on enzymatic degradation of plant cell walls and applied it for extraction of EVs from MO roots. To prevent the contamination with intercellular vesicles during the experiment, plant tissues were washed multiple times, protoplast protector was added, and enzymatic digestion time was shortened maximally. TEM revealed that the extracted MOEVs had a very similar morphology and structure to mammalian exosomes, were largely intact, and had a uniformly small diameter (50–80 nm) (Fig. 2A and B), corroborating the results reported by literature^1, 35^. The multivariate statistical analysis screened 182 differential metabolites between MOEVs and MONVs, which were mainly enriched in metabolic pathways related to the functions of EVs, such as RNA transport, autophagy–other, and endocytosis. It is not surprising that EVs are enriched in these pathways, as they have been shown to perform these functions^31, 36–41^. The modified methodology for EV extraction yielded more EVs with higher purity compared to NVs extracted by conventional juicing.

MOEVs differ from MONVs in terms of lipid, RNA, and protein composition. In comparison, MOEVs contained less RNA, more protein species, and greater protein content. Non-targeted metabolomics analysis showed that both vesicles had similar types of compounds and their amounts, but the relative amounts of compound types differed significantly. Both MOEVs and MONVs contained high amounts of lipids (87.62% vs 66.17%, respectively), mainly PC, PE, PS, PI, phosphatidylglycerol, and phosphatidic acid. The greater content and size of lipids in MOEVs may facilitate the transfer of EVs across the membrane to reach cell wall. The lipids of plant EVs may themselves act as signaling molecules as they accumulate in response to pathogen infection and stress, and they play an important role in immunomodulatory defense^36^. Lipid composition was significantly different between MOEVs and MONVs. MOEVs contain mostly PC (46.40%), PE (29.06%), and PS (7.71%), whereas MONVs contain mostly PC (29.18%), PE (14.86%), and PI (8.53%). These differences suggest that MONVs are more complex than MOEVs, and we speculate that they likely originate from mixed particles.

MOEVs and MONVs differ in particle size, protein, RNA, and lipid composition, and their role in promoting miR-155 expression in ECs. Recent studies have highlighted the important role of blood vessels in osteogenesis^42,43^, and an increasing number of studies have reported that blood vessels are closely associated with osteogenesis in aged and de-ovulated mice with osteoporosis, where the number of H-type vessels is significantly reduced^1, 44–46^. miR-155 regulates neovascularization and osteogenic differentiation. Recent studies have shown that ECs can also secrete exosomes that specifically target bone tissue and improve osteoporosis by delivering miR-155 in vitro and in vivo^47^. Therefore, promoting high miR-155 expression in ECs is considered an effective therapeutic strategy to improve osteoporosis. Our study showed that MOEVs could slightly inhibit miR-155 expression in endothelial cells (negative correlation) at 24 h, while substantially promoting miR-155 expression in endothelial cells in a concentration-dependent manner at 48 h. It suggests that anti-osteoporotic effect of MOEVs may be around 48 h after 24 h of drug ingestion, although we do not have enough experimental data to determine the exact mechanism behind this phenomenon. Our study also showed that MONVs at a concentration of 0.5 × 10^9^ particles/mL strongly promoted miR-155 expression in endothelial cells at 48 h, which was 2.5-fold higher than that obtained by the same concentration of MOEVs; however, the effect did not increase at a higher concentration of 4.5 × 10^9^ particles/mL. Apparently MOEVs are a subgroup of MONVs, but MOEVs in mixed particles could not resolve this phenomenon. There is clearly another type of particle that is distinct from MOEVs. This particle is likely an intercellular vesicle that may have better biological activity at lower and safer concentrations. Although methods to isolate subpopulations of particles are still limited, this still raises extensive interest in their subpopulations. This data suggests that we cannot ignore the possibility of intercellular vesicles as therapeutically active agents and drug carriers. Protoplasts prepared by enzymatic cell wall digestion can be used to further study intercellular vesicles.

KEGG analysis of the differential metabolites showed that MOEVs and MONVs have different biological functions. In addition to RNA transport, autophagy (autophagy–other), and endocytosis, other main functions include linoleic acid metabolism, phenylalanine metabolism, phenylpropanoid biosynthesis, sphingolipid metabolism, and purine metabolism. These important differences in biological pathways suggest that these two particles play different biological roles in the growth and development of MO.

It is important to note that, because the applied enzymatic degradation of cell walls for the extraction of protoplasts successfully isolated and extracted plant derived EVs, we did not further explore the optimal experimental conditions. This method must be adapted to the specific plant species and tissue type by adjusting the type, ratio, and enzyme digestion time, as well as by optimizing the pH and adding cytoprotectants if necessary.

## Conclusion

Our newly developed method for EVs extraction from plants by degradation of the cell wall can be used to extract large amounts of EVs. The results suggest that MOEVs are candidates for novel natural active substances and drug carriers that can be elevated by promoting miR-155 expression in endothelial cells. MONVs and MOEVs are distinct particles different in size, biological activity, and contents. We propose that MOEVs can be used as active substances or drug carriers for the treatment of orthopedic diseases. Further animal experiments should be conducted to verify their biological activity and toxicity. High yield, easy standardization, and enzyme recovery render this method of enzymatic cell wall degradation suitable for preparation of plant-derived extracellular vesicles.

## Materials and methods

### Animals and cell lines

Wild-type C57BL/6 mice were purchased from Spelford Biotechnology Co. (Beijing, China). All animal experimental protocols were approved by the Experimental Animal Ethics Committee of the Ruiye Model Animal Center. A mouse brain-derived endothelial cell line (bEnd.3) and human umbilical vein endothelial cells (h-UVECs) were purchased from Shanghai Meiwan Biotechnology Co. (Shanghai, China). Cells were cultured in dulbecco’s modified eagle medium high sugar medium with 10% fetal bovine serum, 100 U/mL penicillin, and 100 mg/mL streptomycin, all purchased from Gibco (Thermo Fisher Scientific, Waltham, MA, USA). Cells were cultured at 37 °C and 5% CO_2_.

### Separation of MONVs and MOEVs

*Morinda officinalis* was obtained from Zhaoqing, Guandong Province, China. Two hundred grams of washed *M.officinalis* roots were squeezed and the obtained juice was filtered (Fig. 1). Differential centrifugation (500 × *g* for 10 min, 2000 × *g* for 20 min, 5000 × *g* for 30 min, and 10,000 × *g* for 60 min) was performed to remove large particles. The pellet was removed from the centrifugation tube after each step. The collected supernatant was centrifuged at 100,000 × g for 70 min. The pellet was resuspended in PBS, and then passed through a 0.22 μm filter. The protein concentration of vesicles was determined using the BCA protein assay kit (Beyotime, Shanghai, China).

To release MOEVs into the solution, plant cell walls were degraded with enzymes following a modified protocol for plant protoplast preparation^24–27^. Briefly, fresh roots of MO were cut into 1 mm pieces using a special slicer for Chinese medicine before enzyme digestion to increase the surface area and accelerate the rate of enzyme degradation of the cell wall. The pieces were washed with PBS to remove intracellular vesicles released from broken cells. The washed pieces were digested with a mixture of vesicle isolation buffer (0.1% MES, 1 mmol/L CaCl_2_, pH 5.5), cellulase R10 and pectinase R10, and hemicellulase (Cangzhou Xiasheng Enzyme Biotechnology Co. Hebei, China) in a 1:1:1 ratio and 0.5% concentration. The solution was gently shaken, and the digest was collected after 2 h following a modified protocol as that used for juice extraction (Fig. 1).

During isolation, samples were kept on ice or stored at 4 °C. After isolation, samples were stored at 4 °C for less than seven days or frozen at − 80 °C. Freeze–thaw was not used more than once. NanoFCM and TEM assays were performed using fresh samples. For metabolomics analysis, all samples were used after freezing once at − 80 °C.

### NanoFCM detection of diameters and concentrations of MONVs and MOEVs

Separated vesicles were assayed for diameter and concentration by NanoFCM, a technique for determining small particle size distribution profiles in suspensions using known standards. Samples were diluted 1:100 and analyzed using a Flow NanoAnalyzer (Xiamen Fuliu Biotechnology Co., Fujian, China) according to the manufacturer’s protocol. The basic procedure was as follows: a 250 nm quality control particle calibration laser was used as a reference for particle concentration. A mixture of particles of different sizes (68–155 nm) were used to determine the reference curve of the diameter distribution, and the sample particle concentration and size distribution were calculated using NF Profession 1.0 (Xiamen Fuliu Biotechnology Co., Fujian, China).

### Transmission electron microscopy detection of MONVs and MOEVs

Sample solution (5–10 µL) was added in a dropwise manner to the copper grid and left to sediment for 3 min. Excess solution was removed with filter paper, and the solution was stained using phosphotungstic acid negative staining after PBS rinsing. The samples were dried at room temperature for 5 min and imaged using TEM (JEM-1200EX, JEOL, Ltd., Tokyo, Japan) with an operating voltage of 80–120 kV.

### Flow cytometric viability assay

h-UVECs were cultured on a proliferation medium. The h-UVECs were inoculated with 2000 cells/μL in 96-well plates and incubated for 12 h. The h-UVECs were treated with MONVs and MOEVs at concentrations of 0.5 × 10^9^ particles/mL and 4.5 × 10^9^ particles/mL for 24 h and 48 h, respectively. h-UVECs were fluorescently dual-labeled (Annexin V and PI) and assayed for viability using a flow cytometer (Mindray, Shenzhen, China) to detect cell viability.

### Cytotoxicity evaluation by CCK-8 assay

h-UVECs were cultured on a proliferation medium. After incubation for 12 h, h-UVECs were treated with MONVs and MOEVs at 0.5 × 10^9^ particles/mL and 4.5 × 10^9^ particles/mL concentrations for 12, 24, 48, and 72 h, and the optical density (OD) values at each time point were measured using an enzyme marker. Time point OD values were obtained using enzyme linked immunosorbent assay.

### Endothelial cell uptake assay

Vesicle entry was monitored for 2, 4, and 8 h after treatment of b. End3 and h-UVECs with MONVs and MOEVs labeled with DiI stain (Invitrogen, Carlsbad, CA, USA). The proliferation medium was removed, and the cells were fixed with 4% paraformaldehyde. Then, Hoechst 33,342 (blue) was added to the cells, and the nuclei were stained by incubation at room temperature for 15 min. Finally, the cells were washed with PBS containing 1% bovine serum albumin, and the fluorescence (red and blue) was imaged under a fluorescence microscope (Leica, Wetzlar, Germany). At least three fields of view were selected and analyzed using ImageJ 1.8.0 software.

### In vivo biodistribution assay

To analyze the biodistribution of MONVs and MOEVs in vivo, healthy C57BL/6 females (7–8 weeks old) were injected intravenously with DiR-labeled MONVs and MOEVs. At 12, 24, and 48 h after injection, the mice were euthanized, and different organs were collected. DiR signal intensity from different organs was measured using an IVIS Lumina III In Vivo Imaging System (PerkinElmer, Waltham, MA, USA) and analyzed with a Living Image in vivo imaging analysis software (PerkinElmer, Waltham, MA, USA).

### RNA extraction and determination of miR-155 expression in h-UVECs

To assess the miR-155 expression in endothelial cells, primers were designed as follows: miR-155 (forward: 5′-TTAATGCTAATTGTGATAGGGGT-3′, reverse: 5′-ACCCCTATCACAATTAGCAT-TAA-3′); U6 (forward: 5′-ATTGGAACGATACAGAGAAGATT-3′, reverse: 5′- GGAACGCTTCACGAATTTG-3′).

Total RNA was extracted from each group of cells using the TRIzol method and reverse transcribed into cDNA using the miRNA First Strand cDNA Synthesis Kit (Sangon Biotech, Shanghai, China). cDNA of miR-155 and internal reference U6 was amplified using SYBR Premix Ex Taq II (CWBio, Jiangsu, China).

### Metabolomics analysis

Equal amounts of each of the samples were mixed as quality control (QC) samples. The QC samples were interspersed between samples during the mass spectrometry loading process. Methanol, formic acid, and acetonitrile were purchased from Chemicals Northwest (Shanghai, China), and L-2-chlorophenylalanine was purchased from Shanghai Hengchuang Biotechnology Co. (Shanghai, China). All extraction reagents were pre-cooled at − 20 °C before use. The samples were first lyophilized, mixed with 400 μL of methanol, vortexed for 30 s, and sonicated for 3 min. Two small steel balls were added to the samples, and the samples were pre-cooled at − 20 °C for 2 min before grinding in a grinder (60 Hz) (Midea, Guangdong, China) for 2 min. The ground samples were centrifuged at 13,000 rpm for 10 min at 4 °C. The supernatant (350 μL) was evaporated into LC–MS injection vial. The sample was re-dissolved in 300 μL aqueous methanol (1:4, v/v), vortexed for 30 s, sonicated for 3 min, and subsequently stored at − 20 °C for 2 h. The sample was centrifuged at 13,000 rpm for 10 min at 4 °C, and 100 μL of the supernatant was aspirated with a syringe, filtered using a 0.22 μm organic phase pinhole filter, and transferred to the LC injection vial for examination. The LC–MS analysis was conducted in a Dionex U3000 UHPLC ultra-high performance liquid tandem QE high-resolution mass spectrometer, consisting of a liquid–liquid mass spectrometer, under the following parameters: chromatographic column ACQUITY UPLC HSS T3 (100 mm × 2.1 mm, 1.8 μm), 45 °C, water (containing 0.1% formic acid) and acetonitrile (containing 0.1% formic acid) as mobile phases, flow rate of 0.35 mL/min, an ESI ion source, and sample mass spectrometry signal acquisition using the positive and negative ion scan modes.

The raw data were processed using metabolomics software Progenesis QI v2.3 software (Nonlinear Dynamics, Newcastle, UK) for baseline filtering, peak identification, integration, retention time correction, peak alignment, and normalization. Compound identification was based on accurate mass numbers, secondary fragmentation, and isotopic distribution. Characterization was performed using The Human Metabolome Database (HMDB: https://hmdb.ca/), Lipidmaps (v2.3: https://www.lipidmaps.org/), and METLIN databases (http://metlin.scripps.edu), as well as self-built libraries. For the extracted data, the ion peaks with missing values (0 values) > 50% within the group were removed and the zero values were replaced by half of the minimum value. The compounds obtained from the characterization were screened according to the scoring of the compound characterization results (Score) with a screening criterion of 36 out of 60; < 36 points were considered inaccurate and deleted.

### RNA, protein, and lipid assays of MOEVs and MONVs

To purify lipids from MOEVs and MONVs, the sample was mixed with chloroform and methanol in a ratio of 3:8:4 (v/v/v) (sample: chloroform: methanol) and centrifuged at 10,000 × *g* for 10 min, after which the sample was divided into three layers, the upper water, middle protein, and bottom lipid layer. The bottom layer was collected, dried at 100 °C, and resuspended with chloroform; the lipids in the samples were detected using TLC.

To determine whether MOEVs and MONVs contain RNA, extracted vesicles were aliquoted into two tubes, and after the addition of QIAzol lysate (QIAGEN, Germany), the tubes were shaken vigorously and incubated for 30 min at room temperature. One tube with RNase A (Solarbio, Beijing, China) and one tube without RNase A were incubated for 30 min at 37 °C, and the RNA was extracted with RNeasy Mini Kit (QIAGEN, Germany). MOEVs and MONVs RNA were detected by 2.5% agarose gel electrophoresis.

Two vesicle suspensions of two different concentrations were prepared in four separate tubes, two of which were with stock solution and two with a solution diluted tenfold. Two of the tubes with different concentrations of vesicles were lysed by adding Radio Immunoprecipitation Assay (RIPA) and Phenylmethanesulfonyl Fluoride (PMSF) (100:1; Solarbio, Beijing, China) lysis solution, and the other two tubes (without any reagent) were used as controls. A 10% SDS-PAGE gel was prepared according to the SDS-PAGE gel kit (Beyotime, Shanghai, China). A sample volume of 20 µg was added in each gel well, and after electrophoresis, the gels were stained according to the instructions of the Fast Silver Stain Kit (Biosharp, Hefei, China) and imaged using a scanner (Epson, Japan).

### Differential metabolite pathway analysis

We performed metabolic pathway enrichment analysis of MONVs and MOEVs differential metabolites using the KEGG database (https://www.kegg.jp/).

### Statistical analyses

Data obtained from the experiments are expressed as mean ± SD. Statistical analyses were performed using one-way analysis of variance (ANOVA) and the *t*-test in GraphPad Prism (GraphPad Prism Software Inc., San Diego, CA, USA). Results were statistically significant at *p* < 0.05, *p* < 0.01, and *p* < 0.001.

In the non-targeted metabolite analysis, unsupervised PCA was first used to observe the overall distribution between samples and the stability of the whole analysis process. Supervised PLS-DA and OPLS-DA were used to distinguish the overall differences in metabolic profiles between groups and to find the differential metabolites between these groups.

In the OPLS-DA analysis, the variable importance in projection (VIP) was used to measure the strength and explanatory power of the expression pattern of each metabolite on the categorical discrimination of each group of samples, and to explore the biologically significant differential metabolites. The *t*-test was further used to verify whether the differential metabolites between groups were significant. The screening criteria were VIP values of > 1 for the first principal component of the OPLS-DA model, and a *p*-value of < 0.05 for the *t*-test.

Correlation analysis was performed using the Pearson correlation coefficient to measure the degree of linear correlation between two metabolites.

**method Statement.** All methods were performed in accordance with the relevant guidelines and regulations. Experimental research and field studies on plants (either cultivated or wild), including the collection of plant material, comply with relevant institutional, national, and international guidelines and legislation.

### Ethics approval

All animal experimental protocols were approved by the Experimental Animal Ethics Committee of the Ruiye Model Animal Center. The study is reported in accordance with ARRIVE guidelines 2.0.

### Supplementary Information

Supplementary Information.

## Data Availability

All data generated or analysed during this study are included in this published article and its supplementary information files.

## Abbreviations

MO: Morinda officinalis
NVs: Nanovesicles
EVs: Extracellular vesicles
b.End3: Mouse brain-derived endothelial cell line
h-UVECs: Human umbilical vein endothelial cells
ECs: Endothelial cells
NanoFCM: Nano-flow cytometry
OD: Optical density
DiI: Lipophilic 1,1′-dioctadecyl-3,3,3′,3′tetra- methylindocarbocyanine perchlorate
DiR: 1,1-Dioctadecyl-3,3,3,3-tetramethylindotricarbocyanine iodide
QC: Quality control
MTX: Methotrexate
LC-MS: Liquid chromatograph-mass spectrometer
HMDB: Human metabolome database
RIPA: Radio immunoprecipitation assay
PMSF: Phenylmethanesulfonyl fluoride
ANOVA: One-way analysis of variance
VIP: Variable importance in projection
NIM: Negative ion mode
PIM: Positive ion mode
PC: Phosphatidylcholine
PBS: Phosphate-buffered saline
HPLC: High performance liquid chromatography
TEM: Transmission electron microscopy
TLC: Thin-layer liquid chromatography
FCM: Flow cytometry
BCA: Bicinchoninic acid assay
CCK-8: Cell counting kit-8
PCR: Polymerase chain reaction
PPI: Protein-protein interaction network
SDS-PAGE: Sodium Dodecyl Sulfate PolyAcrylamide gel electrophoresis
PLS-DA: Partial least-squares discriminant analysis
OPLS-DA: Orthogonal partial least-squares discriminant analysis
KEGG: Kyoto encyclopedia of genes and genomes
