# Supplementary material for: An enzyme-based system for extraction of small extracellular vesicles from plants
Source: Sci Rep. 2023 Aug 25;13:13931. doi: 10.1038/s41598-023-41224-z (PMC10457285; doi:10.1038/s41598-023-41224-z)
Supplement: Supplementary file 1 — Supplementary Information. [file 41598_2023_41224_MOESM1_ESM.docx]

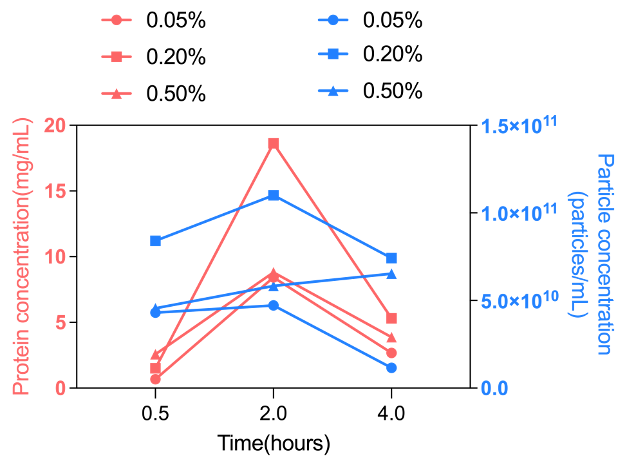


**Supplementary Figure 1:** Mapping the optimum enzyme reaction concentration and reaction time using nanoFCM (n=3).


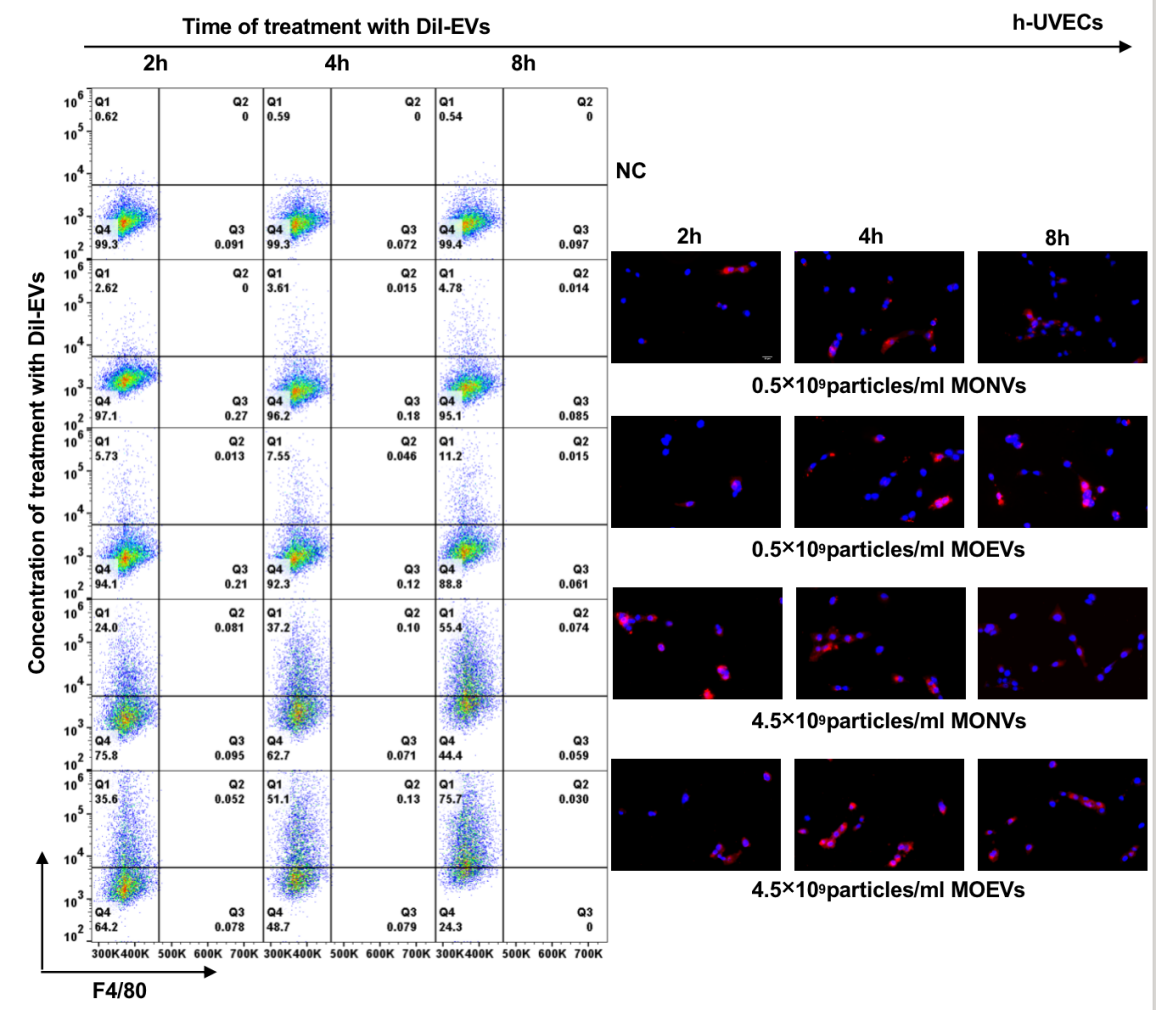


**Supplementary Figure 2:** Flow cytometry assays and fluorescent microscopic images about Dil-labeled MONVs and MOEVs of different concentrations (0.5 × 10^9^ particles/ml, 4.5 × 10^9^ particles/mL) uptaken by h-UVECs.

***
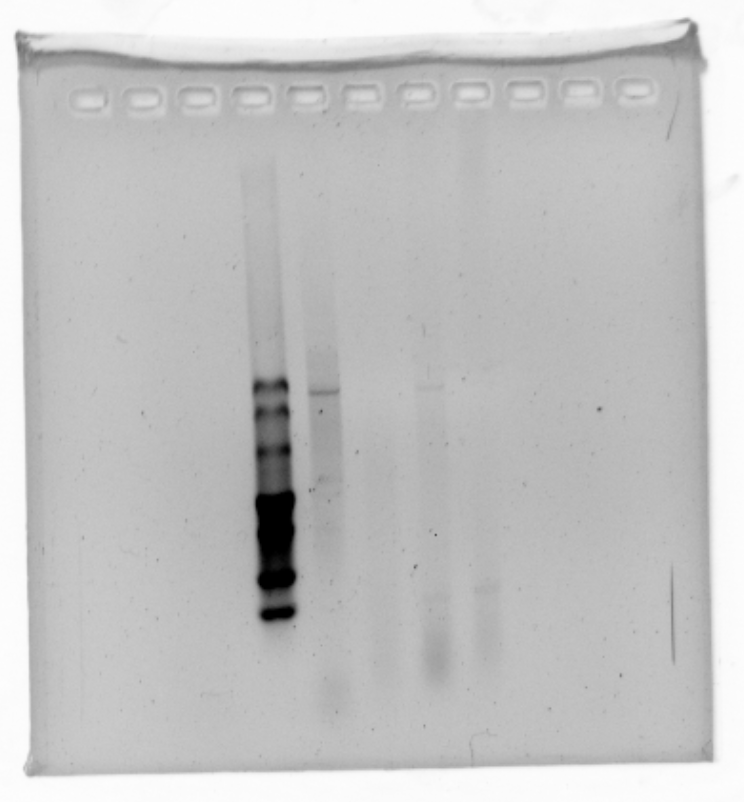

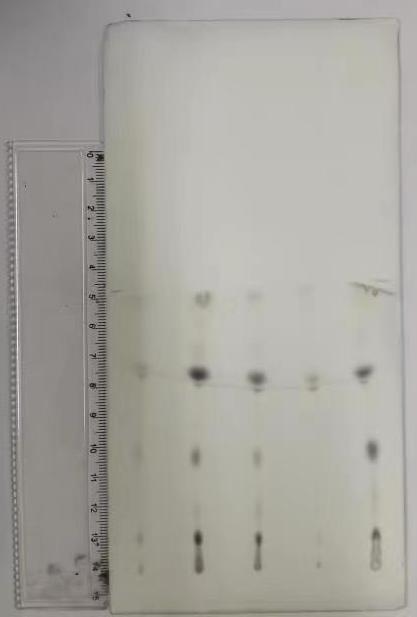
***

***
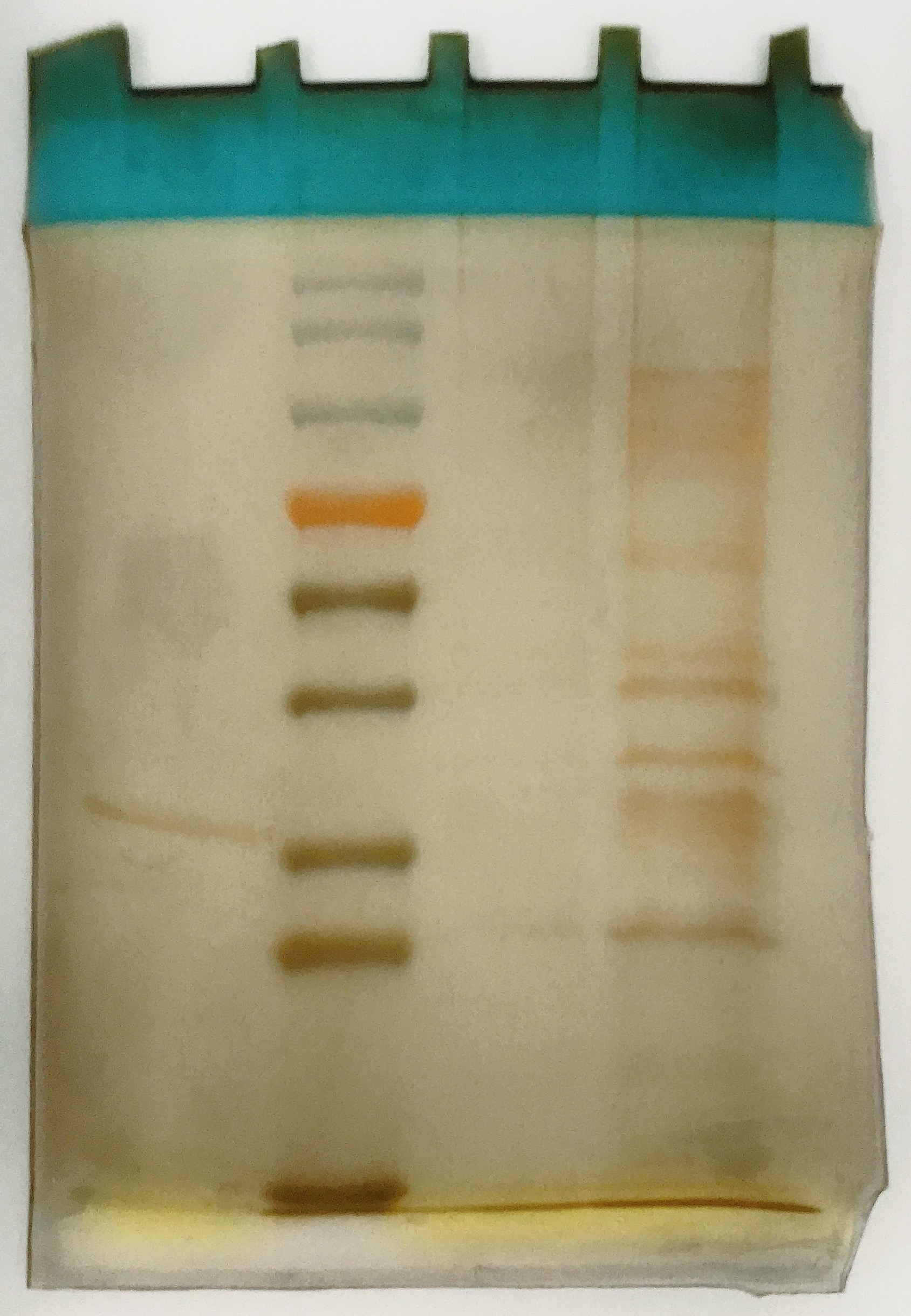

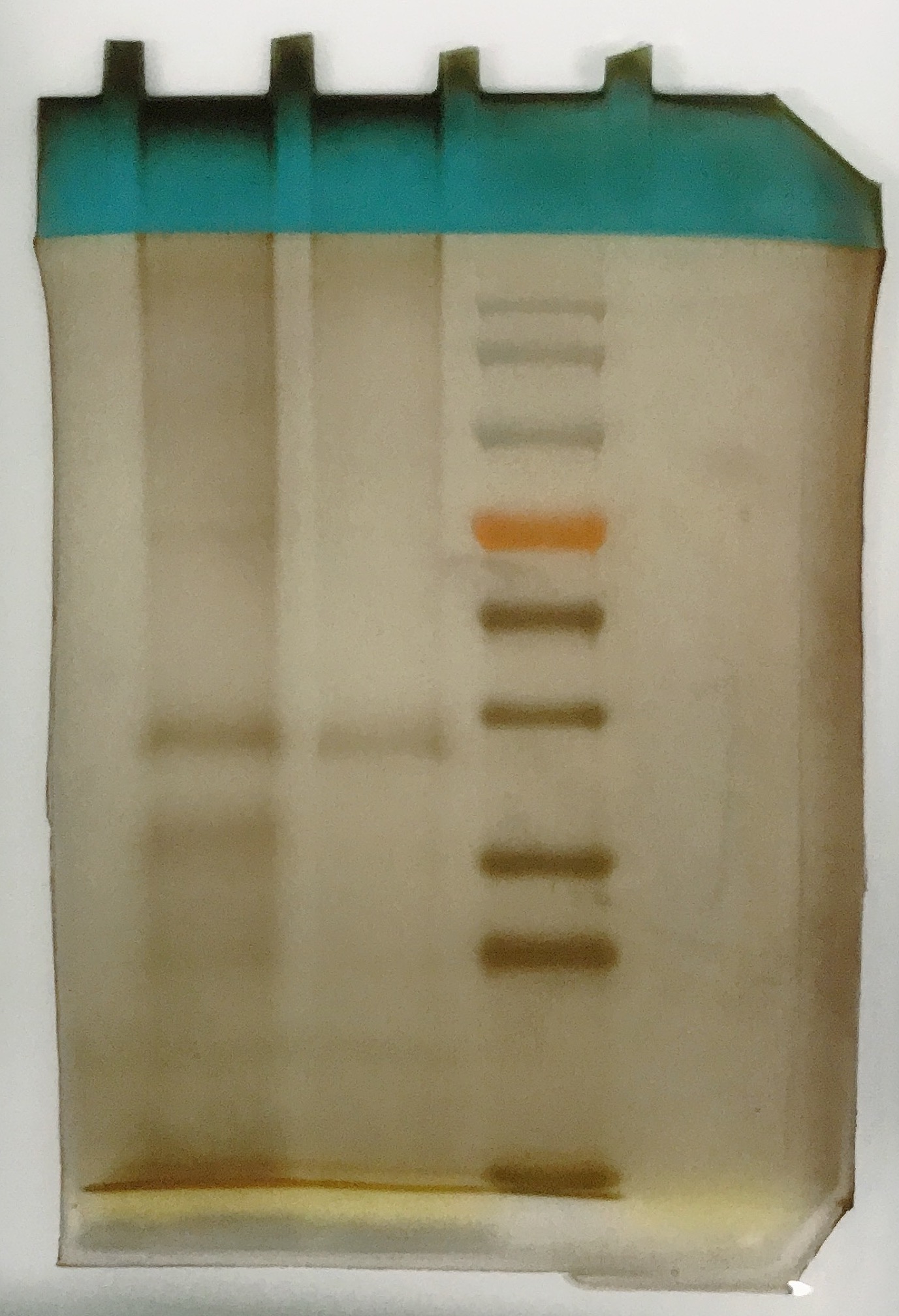
***

**Supplementary Figure 3:** The original, unprocessed versions of **Figure 2G、H and I**.
